# Religiosity, Telomere Length and Preterm Birth Among Mexican and African American Women in Texas

**DOI:** 10.1007/s10943-025-02500-6

**Published:** 2025-11-22

**Authors:** Weiyi Huang, Amy M. Burdette, Gang Han, Brandie DePaoli Taylor, Gabriel Neal, Robin L. Page

**Affiliations:** 1 Department of Biostatistics and Data Science, University of Texas Health Science Center at Houston, Houston, TX USA; 2 Department of Sociology and Public Health Program, Florida State University, Tallahassee, USA; 3 Department of Epidemiology and Biostatistics, Texas A&M University, College Station, TX USA; 4 Advocate Aurora Research Institute, Milwaukee, WI USA; 5 Department of Primary Care and Rural Medicine, Texas A&M College of Medicine, Bryan, TX USA; 6 College of Nursing, Texas A&M University, College Station, TX USA

**Keywords:** Religiosity, Telomere, Preterm birth, Hispanic health

## Abstract

We conducted a cross sectional study of 175 pregnant women (100 Mexican and 75 African American women) recruited from three prenatal clinics in Central and East Texas. This study explored the relationships between religiosity, telomere length, and preterm birth. Results indicated a significant association between telomere length and birth outcomes, with shorter telomeres observed in preterm births compared to full-term births. Mexican women attended religious activities more frequently, while African American women tended to rely on religious or spiritual beliefs to address daily challenges. Mediation analysis revealed that more frequent religious service attendance showed an association with lower preterm birth risk via telomere length on the risk-difference scale of 0.03 (95% CI 0.001 to 0.079; *P* = 0.04) when comparing women who never attend with those who attend every week or more. Higher self-reported religiosity showed an association with lower preterm birth risk independent of telomere length on the risk-difference scale of 0.17 (95% CI 0.036 to 0.309; *P* = 0.02) when comparing women who are not religious at all with those who are very religious. Future research is needed to investigate the complex interactions among these factors using longitudinal designs and larger sample sizes, to inform the development of effective strategies for preventing preterm birth.

## Introduction

Preterm birth, defined as a live birth occurring before reaching 37 weeks of gestation, had a global prevalence of approximately 9.9% in 2020, with an estimated 13.4 million newborns affected (Ohuma et al., 2023). Preterm birth, as one of the main risk factors for neonatal mortality under 5 years of age, remains a significant global public health challenge (Ohuma et al., 2023). Preterm birth rates continue to reflect complex disparities influenced by maternal race, ethnicity, and nativity, with Black women facing disproportionately higher risks of extreme prematurity as compared to other racial groups (Egbe et al., 2022). African American women face significant barriers to accessing quality healthcare and living in well-resourced communities as compared to non-Hispanic white women, which may exacerbate the risk of preterm birth (Giurgescu et al., 2022). Conversely, Hispanic women have similar rates of preterm births to non-Hispanic white women (10.06% vs. 9.44%) despite lower socioeconomic status (Osterman et al., 2024).

*Race, Ethnicity, Telomere Length, and Preterm Birth*

Previous research has revealed important links between race, ethnicity and biological functioning. Chronic stress resulting from racial and other social inequalities significantly disrupts biological processes, including DNA damage, telomere maintenance, mitochondrial dysfunction, and inflammation, thereby accelerating cellular aging (Polsky et al., 2022). Cellular senescence is crucial for parturition and decidual senescence is proposed to be related to spontaneous preterm labor (Gomez-Lopez et al., 2017). Telomere length is a commonly used biomarker of cellular aging (Blackburn et al., 2015). Telomeres are protective caps located at the ends of chromosomes that naturally shorten with cell division, and their length is influenced by various factors including stress and environmental exposures (Shalev et al., 2013). Telomere shortening has been associated with numerous adverse health outcomes, including diabetes, myocardial infarction, cancer, and accelerated aging (Blackburn et al., 2015). A recent review suggested that the risk factors for preterm birth among African American women may be reflected by epigenetic telomere shortening itself (Phillippe, 2022). Mexican women are also often affected by structural inequalities, yet they have lower preterm birth rates than Black women. Findings for birth outcomes among Hispanic women are consistent with a phenomenon commonly referred to as the “Hispanic Health Paradox” (Markides and Eschbach, 2005). The Hispanic paradox refers to consistent findings that Hispanics, as a group have mortality outcomes equal or surprisingly better than non-Hispanics in the United States, despite ranking low on most indicators of socioeconomic status (Franzini et al., 2001). A recent cohort study of in-hospital live singleton births in the US by Barreto and colleagues (Barreto et al., 2024) further displays this trend with showing non-US born Hispanic mothers having among the lowest rate of pre-terms births as compared to all other racial and ethnic groups.

*Race, Ethnicity, and the Influence of Religious Involvement on Telomere Length & Preterm Birth*

Researchers have hypothesized that religion may be part of the reason for lower mortality rates among Hispanics as compared to other racial and ethnic minorities. Higher levels of religious involvement may buffer stress among Mexican women and contribute to their relatively favorable health outcomes (Moreno and Cardemil, 2018). Similarly, research has documented salutary effects of religious attendance on health among African Americans, particularly women (Ellison et al., 2010).

Scholarship examining the relationship between religiosity and leukocyte telomere length suggests that religious involvement protects against cellular aging (Hill et al., 2016). Subsequent work by Hill, Hill et al. (2017) proposes that religious service attendance indirectly influences telomere length by reducing symptoms of depression and risk of smoking. To date the relationship between religiosity and telomere length among pregnant women remains unexplored. Similarly, research on religion and telomere length has centered on majority non-Hispanic white populations.

Research focusing on maternal religious involvement suggests the health benefits of religious involvement may extend across generations (from mother to child), as mothers who attend religious services regularly are less likely to deliver a low-birth-weight infant (Burdette et al., 2012). Religious attendance has also been linked to breastfeeding initiation and duration (Burdette and Pilkauskas, 2012; Stroope et al., 2018). The association between various forms of religious involvement and preterm birth has yet to be examined.

Why might religious involvement be associated with lower rates of preterm birth? Previous research suggests that religion may improve birth outcomes by improving mental health and reducing negative health behaviors. Studies show that religious attendance is associated with better mental health across a range of indicators, psychological distress (Hill et al., 2011). Religious involvement benefits mental health by promoting social (e.g., social support) and psychological (e.g., optimism and a sense of meaning and purpose) resources (Koenig et al., 2001). Research also suggests that poor mental health is a significant risk factor for several negative birth outcomes, including preterm birth (Rondó et al., 2003). Social support and coping mechanisms, such as religiosity, may play a positive role in mitigating stressors, improving mental health and birth outcomes (Bjørlykhaug et al., 2021; Burdette et al., 2012; Page et al., 2009). In addition, exposure to stressors has been shown to influence telomere length (Polsky et al., 2022), suggesting a biological pathway through which stress may affect cellular aging and health outcomes.

Religious attendance might also protect against preterm birth by encouraging positive health behaviors and discouraging unhealthy lifestyle choices (Jesse et al., 2006; Jesse and Reed, 2004; Magaña and Clark, 1995; Najman et al., 1988; Page, 2004; Page et al., 2009). Studies show that religious attendance is associated with a wide range of healthy behaviors including lower levels of smoking and drinking, and greater use of preventive health care services, like prenatal (Hill et al., 2007, 2011; Koenig et al., 2001). Research concerning the health behaviors of pregnant and postpartum women confirms that regular religious attendance is associated with lower rates of alcohol use, cigarette use, and illicit drug use (Mann et al., 2007; Page et al., 2009)).

*Conceptual Model*

This study aims to explore the relationships among religious beliefs, telomere length, and preterm birth to gain a deeper understanding of the racial disparities in preterm birth and the underlying social and psychological mechanisms involved. While limited research has examined various parts of the conceptual model presented below, scholarship to date has yet to connect these important concepts. We are examining racial and ethnic subgroup variation in these concepts because religion may operate differently for Black and Hispanic pregnant women. Historically Black churches often have programs and services tailored toward coping with adversity in society (Ferraro and Kim, 2014). In addition, Black churches play a critical role in the social, political, and organizational lives of Blacks and provide instrumental support to members. This may be because Black churches are one of the few American institutions that are owned and operated by African Americans, thus providing a haven from the broader iniquities of society (Pattillo-McCoy, 1998). In comparison, a key aspect of Latino spiritually is its fascination with physical and mental healing. One religious symbol uniquely central to Mexicans and Mexican Americans is the Virgin of Guadalupe (Magaña and Clark, 1995). Magana and Clark (Magaña and Clark, 1995) suggested that the positive attitudes of many Mexican American women toward their pregnancies are strongly tied to this religious image. While it is unclear how the diverse roles of predominately Black and Hispanic religious institutions translate into differential impacts of religious involvement on telomere length and preterm births, the variation these religious institutions warrants examining subgroup variation.

**Fig. 1:**
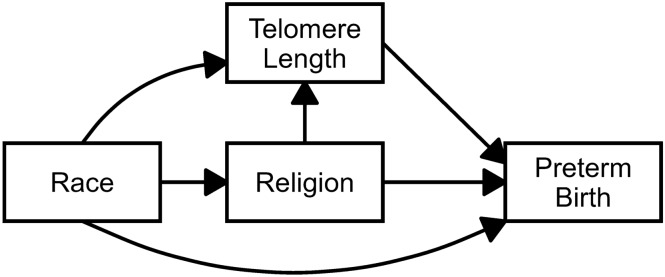
Conceptual Model of Race, Religion, Telomere Length, and Preterm Birth

As shown in Fig.1, race is hypothesized to influence both religious beliefs and telomere length, while religion may affect telomere length, potentially reflecting its role in buffering stress-related telomere shortening. Telomere length, in turn, is posited to impact preterm birth risk. Additionally, race may also have an impact on preterm birth, capturing unmeasured structural or environmental influences. Together, this conceptual model highlights a pathway through which racial background may shape preterm birth outcomes via both psychosocial and biological processes.

## Materials and Methods

### Study Population

We conducted a cross sectional observational study using data from two exploratory, prospective pilot studies. The first study recruited Mexican pregnant women between September 2017 and July 2018, while the second study recruited African American pregnant women between September 2018 and February 2023. These women, aged between 18 and 41 years and at gestational ages ranging from 17 to 39 weeks, were patients at three prenatal clinics in Central and East Texas. Participants self-identified as either Mexican or Mexican American (first study), or as African American or Black (second study). Exclusion criteria included in vitro fertilization, multiple pregnancies, plans to transfer care before delivery, and inability to complete the survey in English or Spanish. All participating women provided written informed consent, and the studies was approved by the university’s Institutional Review Board (IRB2017–0225D & IRB2018–0407D). Participants completed a paper questionnaire and provided a tube of whole blood for telomere analysis. There are totally 175 cases included in this study, with 100 Mexican women and 75 African American women.

### Measures

#### Preterm Birth/Full-term Birth

The duration of pregnancy was determined retrospectively through medical record review after participants had given birth. Gestational length was classified as follows: preterm, less than 37 weeks; and full-term, 37 weeks 0 days to 40 weeks 6 days, in accordance with the American College of Obstetricians and Gynecologists (ACOG, 2012).

#### Telomere Length

First, within 24 h of collection, whole blood was centrifuged to separate the plasma portion, and the buffy coat (containing white blood cells) was collected and stored at -$80^\circ$C until all participants were enrolled. Subsequently, samples were transported to the institution’s core genomics facility, where they were stored at -$80^\circ$C until leukocyte isolation, DNA extraction, and telomere length analysis began. DNA was extracted from the buffy coat by washing the leukocyte pellet to remove circulating free DNA, ensuring that telomere length analysis reflected intact maternal cells. Telomere length was assessed using monochrome multiplex quantitative real-time polymerase chain reaction (qPCR), following the method described by Cawthon (Cawthon, 2009). Each sample was amplified in triplicate on two 384-well plates, with standard curves and negative controls included. Amplification data were analyzed using CFX Maestro software to generate quantitative data. The relative ratio of telomere to single-copy gene (T/S) was calculated according to Cawthon’s method (Cawthon, 2009). Samples exhibiting an intra-assay coefficient of variation (CV%) > 10% across the three replicates or an inter-plate CV% > 10% were re-run within two days, and if further re-running was necessary, fresh DNA aliquots were used.

#### Religiosity

Religiosity was measured through responses assessing three domains: overall religiosity, religious support, and religious coping (Idler et al., 2003). The assessment of religiosity included questions such as “How religious are you?” with higher scores indicating greater religiosity (1 = Not religious at all, 3 = Very religious). It also included questions on the frequency of attending religious services (1 = Never, 7 = Every week or more), the frequency of prayer (1 = Never, 6 = Several times a day), and the frequency of turn to religious beliefs to help deal with daily problems (1 = Never, 5 = Always), with higher scores indicating higher frequency. The religious support domain included questions on the frequency of interaction with church members (1 = Never, 6 = Nearly every day) and of receiving help from the church (1 = Never, 4 = Very Often), with higher scores indicating more frequent support. Religious coping was assessed with statements such as, “When good or bad things happen, I see it as a part of God’s plan for me.” “God has decided what my life shall be.” and “I depend on God for help and guidance.” Higher scores indicated stronger agreement with these statements (1 = Strongly disagree, 4 = Strongly agree). These items were combined into a composite religious coping score using the average value, based on internal consistency analysis. These measures have been documented in the previous study on religion and health (Hill et al., 2016).

### Statistical Analyses

Multiple imputation was applied to deal with the missing values (van Buuren and Groothuis-Oudshoorn, 2011). Descriptive statistics included sociodemographic characteristics (i.e., age, race, marital status, education level, employment status, and household income), telomere length and religiosity. Categorical variables were reported as frequencies and percentages, while continuous variables were reported as medians and interquartile ranges (IQR). The Mann–Whitney U test and Fisher’s exact test were used to compare telomere length and sociodemographic characteristics across different birth outcomes and racial groups. Gestational length was treated as a binary variable (preterm vs. full-term birth). Each aspect of religiosity between Mexican and African American women was compared using the Mann–Whitney U test. For each religiosity item, treated as an ordinal categorical variable, differences between African American and Mexican women were tested using the Cochran–Armitage trend test; p values across the comparisons were adjusted using the Benjamini–Hochberg procedure (reported as q values). Mediation analysis combined with Quasi-Bayesian sampling (Tingley et al., 2014) were used to analyze the impact of telomere length on the relationship between each aspect of religiosity and preterm birth. A p value of less than 0.05 was considered significant. Statistical analyses were performed using R statistical software version 4.4.0.

## Results

Telomere length was significantly associated with preterm birth (Table 1). Specifically, those exhibiting shorter telomeres displayed a greater risk of a preterm birth than those exhibiting lowers telomere length (median T/S ratio 188.0 vs 217.0). No significant difference in telomere length was found between Mexican and African American participants (*p* = 0.46). The median age of the overall sample was 27.0 years; however, there was significant age variation across race (*p* < 0.01), with Mexican participants having a median age of 28.5 years compared to African American participants, who had a median age of 24.0 years. Marital status showed a significant association with race (*p* < 0.01). Among Mexican participants, 69.0% were married compared to only 14.7% of African American participants. Similarly, educational attainment varied across racial lines (*p* < 0.01), with 42.7% of African American participants having more than a high school education versus 19.0% of Mexican participants. Employment status was significantly associated with race (*p* < 0.01), with 49.3% of African American participants being employed compared to 25.0% of Mexican participants. Notably, these sociodemographic characteristics were not associated with birth outcomes.

**Table 1:** Sociodemographic characteristics and telomere length by birth outcomes and race/ethnicity (N=175)

		By birth outcome			By race/ethnicity		
	Total	Full-term	Preterm	*p*-value	Mexican	African	*p*-value
	n (%)	n (%)	n (%)		n (%)	n (%)	
*Age*	27.0	26.0	29.0	0.55	28.5	24.0	<0.01
Median [IQR]	[22.5, 31.5]	[23.0, 31.0]	[22.0, 33.0]		[24.8, 33.0]	[22.0, 29.0]	
Telomere length	214.5	217.0	188.0	<0.01	218.0	213.0	0.46
Median [IQR]	[194.3, 232.6]	[197.0, 237.0]	[174.0, 201.0]		[184.0, 253.0]	[201.0, 226.0]	
*Race*				0.24			–
1. Mexican	100 (57.1)	95 (58.6)	5 (38.5)		–	–	–
2. African American	75 (42.9)	67 (41.4)	8 (61.5)		–	–	–
*Marriage*				1.00			<0.01
1. Married	80 (45.7)	74 (45.7)	6 (46.2)		69 (69.0)	11 (14.7)	
2. Unmarried	95 (54.3)	88 (54.3)	7 (53.8)		31 (31.0)	64 (85.3)	
*Education*				0.53			<0.01
1. High school or less	124 (70.9)	116 (71.6)	8 (61.5)		81 (81.0)	43 (57.3)	
2. More than high school	51 (29.1)	46 (28.4)	5 (38.5)		19 (19.0)	32 (42.7)	
*Employment*				0.55			<0.01
1. Employed	62 (35.4)	56 (34.6)	6 (46.2)		25 (25.0)	37 (49.3)	
2. Unemployed	113 (64.6)	106 (65.4)	7 (53.8)		75 (75.0)	38 (50.7)	
*Household income*				0.75			0.73
1. 20,000 or less	129 (73.7)	120 (74.1)	9 (69.2)		75 (75.0)	54 (72.0)	
2. More than 20,000	46 (26.3)	42 (25.9)	4 (30.8)		25 (25.0)	21 (28.0)

**Table 2:** Comparison of religiosity between Mexican and African American women

Survey Questions	Mexican	African American	*p*-value
Part 1 Religious Support (median score [IQR])			
How often do you attend religious services?	4.5 [3.0, 6.0]	3.0 [2.0, 4.5]	<0.01
How often do you pray?	5.0 [4.0, 5.0]	5.0 [4.0, 6.0]	0.07
How religious are you?	2.0 [2.0, 2.0]	2.0 [2.0, 2.0]	0.33
How often do you see, write, or talk on the telephone with members of your church?	2.0 [1.0, 4.0]	1.0 [1.0, 4.0]	0.49
How often do people in your church help you out?	2.0 [1.0, 3.0]	2.0 [1.0, 3.0]	0.79
How often do you turn to religious or spiritual beliefs to help you deal with your daily problems?	3.0 [2.0, 4.0]	4.0 [3.0, 4.0]	<0.01
Par 2 Religious Coping (median score [IQR])*	3.7 [3.0, 4.0]	3.7 [3.0, 4.0]	0.46

*Composite religious coping score is the average score of three items: When good or bad things happen, I see it as a part of God’s plan for me. God has decided what my life shall be. I depend on God for help and guidance. Each item was rated on a 4-point scale (1 = Strongly disagree, 4 = Strongly agree)

**Fig. 2:**
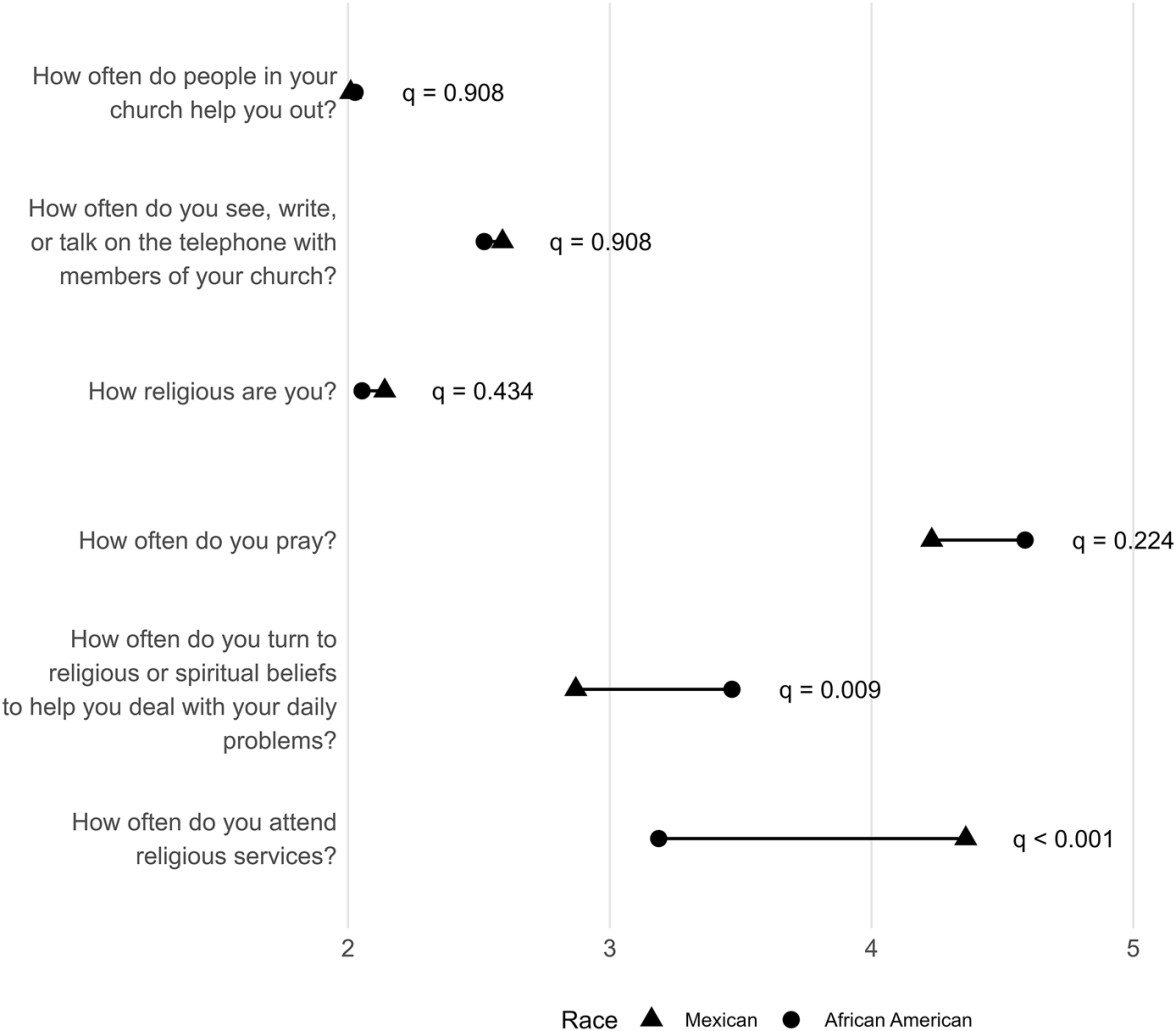
Dumbbell plot of average frequency rating by race/ethnicity (higher = more frequent)

There were distinct patterns of religiosity across racial groups (Table 2). Mexican women reported a significantly higher frequency of religious attendance compared to African American women (*p* < 0.01), while African American women were more likely to turn to religious or spiritual beliefs to address daily problems (*p* < 0.01). However, when comparing African American and Mexican women, no statistically significant differences were observed regarding frequency of prayer, self-reported religiosity, interactions with church members, receiving help from church members, or religious coping. These findings were consistent with the ordinal trend tests (Fig. 2): after Benjamini–Hochberg adjustment, only religious service attendance (Mexican > African American; q < 0.001) and turning to religious/spiritual beliefs (African American > Mexican; *q* = 0.009) remained significant; other aspects of religiosity were not.

We conducted mediation analysis to examine the association via telomere length and the association independent of telomere length between religiosity measures and preterm birth (Table 3). The results showed that higher frequency of religious services attendance was associated with lower preterm birth risk via telomere length and higher self-reported religiosity was associated with lower preterm birth risk independent of telomere length. After adjusting for age and race, comparing women who never attend religious services with those who attend every week or more, the association between religious service attendance and preterm birth outcome via telomere length was 0.03 (95% CI 0.001 to 0.079; *P* = 0.04) on the risk-difference scale and the association between religious service attendance and preterm birth outcome independent of telomere length was not significant in this model. Comparing women who reported they are not religious at all with those who are very religious, the association between self-reported religiosity and preterm birth outcome independent of telomere length was 0.17 on the risk-difference scale (95% CI 0.036 to 0.309; *P* = 0.02), adjusting for age and race. There was no significant association between self-reported religiosity and preterm birth outcome via telomere length. Other aspects of religiosity, including frequency of prayer, interactions with church members, receiving help from church members, turning to religious or spiritual beliefs for daily problems, religious coping, did not show any significant association with preterm birth via telomere length or independent of telomere length.

**Table 3:** Associations among religiosity, telomere length, and preterm birth outcomes

**Survey Questions**	Contrast*	Effect**	**Estimate**	**95% CI**	***p value***
How often do you attend religious services?	Min vs. Max	ACME	0.03	0.001, 0.079	0.04
		ADE	<0.01	$-$0.122, 0.126	>0.99
	per 1-level increase	ACME	0.01	0.0002, 0.013	0.03
		ADE	<0.01	$-$0.021, 0.018	>0.99
How often do you pray?	Min vs. Max	ACME	0.02	$-$0.010, 0.054	0.25
		ADE	0.04	$-$0.116, 0.155	0.44
	per 1-level increase	ACME	<0.01	$-$0.003, 0.016	0.25
		ADE	0.02	$-$0.018, 0.060	0.45
How religious are you?	Min vs. Max	ACME	<0.01	$-$0.041, 0.039	0.99
		ADE	0.17	0.036, 0.309	0.02
	per 1-level increase	ACME	<0.01	$-$0.022, 0.027	0.96
		ADE	0.12	0.022, 0.250	0.01
How often do you see, write, or talk on the telephone with members of your church?	Min vs. Max	ACME	0.02	$-$0.009, 0.058	0.21
		ADE	0.02	$-$0.089, 0.160	0.76
	per 1-level increase	ACME	<0.01	$-$0.002, 0.011	0.22
		ADE	<0.01	$-$0.020, 0.024	0.76
How often do people in your church help you out?	Min vs. Max	ACME	<0.01	$-$0.036, 0.030	0.83
		ADE	0.02	$-$0.091, 0.168	0.76
	per 1-level increase	ACME	<0.01	$-$0.012, 0.010	0.82
		ADE	0.01	$-$0.030, 0.053	0.75
How often do you turn to religious or spiritual beliefs to help you deal with your daily problems?	Min vs. Max	ACME	$-$0.01	$-$0.045, 0.021	0.54
		ADE	$-$0.05	$-$0.198, 0.077	0.46
	per 1-level increase	ACME	<0.01	$-$0.010, 0.005	0.53
		ADE	$-$0.01	$-$0.038, 0.021	0.44
Religious coping***	Min vs. Max	ACME	$-$0.04	$-$0.114, 0.014	0.19
		ADE	$-$0.12	$-$0.423, 0.063	0.30
	per 1-level increase	ACME	$-$0.01	$-$0.021, 0.003	0.18
		ADE	$-$0.02	$-$0.043, 0.026	0.29

*Contrast: Min vs. Max means lowest score vs. highest score; per 1-level increase means local contrast at the sample median score vs. next higher level; models adjusted for age and race. **ACME: average mediation effect/indirect effect, ADE: average direct effect. ***Religious coping: Low = composite religious coping score below the sample median; High = composite religious coping score at or above the sample median

We summarized telomere length as median and interquartile range across categories of each religiosity indicator in the overall sample and by race/ethnicity (Table 4). At the 0.05 level, we observed no statistically significant differences in telomere length across categories for any indicator of religiosity, either overall or within racial/ethnic strata (all *P* > 0.05). However, in the overall sample, the median values of telomere length were generally higher among participants reporting greater religious engagement (for example, attending services, praying, interacting with church members, receiving help, and turning to religious or spiritual beliefs). Within racial/ethnic strata, patterns were inconsistent, likely due to limited sample sizes in some strata.

**Table 4:** Telomere length across religiosity indicators, overall and stratified by race/ethnicity

Survey Questions	scores	n	Overall Median [Q1, Q3]	Mexican Median [Q1, Q3]	African American Median [Q1, Q3]
Part 1: Religious Support*					
How often do you attend religious services?	Never	22	214.0 [201.0, 236.8]	250.0 [224.6, 284.3]	210.9 [200.8, 223.9]
	Ever	153	214.5 [191.5, 231.7]	216.2 [184.3, 252.6]	214.4 [199.9 226.3]
How often do you pray?	Never	10	201.4 [183.2, 245.2]	240.5 [174.5, 268.5]	188.3 [184.4 196.5]
	Ever	165	214.7 [196.4, 231.7]	216.4 [184.8, 252.7]	214.4 [201.2, 226.8]
How religious are you?	Not at all	17	215.7 [200.7, 226.4]	238.2 [173.3, 252.7]	211.3 [201.2, 219.8]
	Moderate/Very	158	214.5 [194.0, 234.1]	216.4 [184.8, 252.7]	213.5 [200.5, 226.9]
How often do you see, write, or talk on the telephone with members of your church?	Never	77	214.5 [199.5, 231.0]	214.5 [190.7, 251.4]	213.5 [202.9, 223.0]
	Ever	98	215.4 [187.9, 239.7]	222.5 [181.1, 253.6]	212.5 [198.5, 226.7]
How often do people in your church help you out?	Never	65	212.5 [188.6, 231.0]	214.5 [166.7, 259.4]	211.6 [201.5, 219.1]
	Ever	110	216.1 [195.4, 236.5]	221.4 [187.3, 248.8]	214.4 [200.7, 227.1]
How often do you turn to religious or spiritual beliefs to help you deal with your daily problems?	Never	29	211.6 [185.0, 252.8]	215.5 [176.3, 257.1]	201.7 [196.1, 212.1]
	Ever	146	215.4 [196.5, 231.3]	219.5 [185.5, 247.4]	214.4 [200.8, 226.9]
Part 2: Religious coping **					
	Low	78	211.7 [196.7, 232.9]	212.5 [186.6, 251.2]	211.3 [200.1, 224.1]
	High	97	216.6 [192.9, 231.7]	222.5 [174.3, 253.6]	215.0 [201.3, 226.8]

Notes: All *P* values > 0.05. * Religious Support: the original scores are dichotomized into Never/Not at all (score = 1) and Ever (score $\ge$ 2). ** Religious coping: Low = composite religious coping score below the sample median; High = composite religious coping score at or above the sample median

## Discussion

This study highlights a significant association between maternal peripheral blood telomere length and preterm birth, with shorter telomere lengths observed in preterm births compared to full-term births. While previous research has examined aspects of our conceptual model, our study is among the first to examine the connections between these concepts using multiple indicators of religious involvement. Previous research in this field is typically limited to one or two measures of religion, typically church attendance or religious salience. In addition, previous research on our focal variables generally centers on non-Hispanic adults in the United States.

Previous studies have revealed that fetal (Schneper et al., 2023; Menon et al., 2012) and maternal (Huang et al., 2024) telomere length show a connection with an increased risk of preterm birth, which may be related to maternal chronic stress (Guzeloglu-Kayisli et al., 2021; Polsky et al., 2022). However, our study showed no direct association between race and telomere length or preterm birth. This may due to our limited sample size, which restricts statistical power (Columb and Atkinson, 2016). Our study found significant differences in patterns of religious participation across racial groups. Mexican women were more likely to attend religious services, while African American women were more likely to turn to religious or spiritual beliefs to handle daily issues. Interestingly, this result contrasts with nationally representative data (Smith et al., 2025). One possible explanation for this discrepancy may lie in the characteristics of our study sample, including the younger average age and lower rates of marriage among African American women, which could influence church involvement. Furthermore, young and single black mothers may experience marginalization or judgment within traditional church spaces, potentially discouraging attendance. Accordingly, the observed racial differences in religious participation likely reflect nuanced social and structural factors that merit more in-depth exploration in future research.

Our mediation analysis revealed that the frequency of religious services attendance was associated with preterm birth outcome via telomere length, suggesting that participation in religious activities may confer biological benefits that reduce the risk of preterm birth. Telomere length, as a biomarker of stress and health status, can be influenced by the psychological and behavioral improvements brought about by religious participation, such as reducing stress or promoting healthy behaviors (Hill et al., 2016). This pathway underscores the potential of religious activities as psychosocial buffers that alleviate stress. Although self-reported religiosity showed an association with preterm birth outcome, the association via telomere length was not significant, indicating that self-reported religiosity may influence birth outcomes through other potential mechanisms. Overall, our results suggests that religious activities may hold potential information to improve interventions centered on maternal health during pregnancy. These findings emphasize the importance of designing targeted interventions within diverse racial and cultural contexts. Our findings contribute to the understanding of complex connections between biological markers, sociodemographic factors, and religious activities intersect to influence preterm birth risk. Interventions aimed at reducing preterm birth rates should consider improving social and spiritual support mechanisms, in addition to addressing broader social inequalities.

Our findings contribute to a growing body of literature examining the relationship between religion and telomere length. Koenig et al. (2016), in a study of female caregivers, found that higher religiosity was associated with longer telomeres, suggesting protective effects against stress-related aging. Hill et al. (2017) extended this by incorporating mediation analysis, revealing that psychosocial pathways may underlie the religiosity–telomere link. In contrast, Ashe et al. (2024) examined religious coping among African American and White adults and found no significant associations between religious coping and telomere length across race or sex groups. Similarly, our study also found no association between religious coping and telomere length or preterm birth. Our study expands on these literature by focusing on pregnant women, incorporating mediation analysis, and considering sociocultural factors that may help explain differences in religiosity and maternal health outcomes.

## Limitations

There are several limitations to the current study. This is a cross sectional study that prevents us from determining the directionality of the associations of religiosity, telomere length, and preterm birth outcomes. While mediation analysis was employed to explore possible pathways, it limits causal interpretation of the mediating role of telomere length. In addition, we employ data from a small sample of pregnant women in Texas. Due to the nature of our sample, our results may not align with national estimates for a variety of reasons. Also, the small sample size reduced statistical power, particularly for stratified analyses. Future research should further examine these associations among diverse populations of pregnant women. The data used in this study did not include several key sociodemographic factors that are related to our core concepts, include immigrant status. This is a notable limitation of our study. Further, the influence of religiosity on telomere length and preterm birth is complex. Any quantitative analysis is likely missing the nuance that would be provided by a qualitative examination of this relationship. Our analysis recommends future scholars consider additional approaches, including qualitative and mixed methodologies.

## Conclusion

This study highlights the complex relationships among religiosity, telomere length, and preterm birth in a racially diverse sample of pregnant women. While religious coping was not significantly associated with telomere length or preterm birth outcome, religious service attendance demonstrated an association with preterm birth outcome via telomere length, and self-reported religiosity also showed an association with preterm birth outcome, independent of the telomere length. These findings underscore the importance of examining multiple dimensions of religiosity in relation to biological and social determinants of health. Future research should employ longitudinal designs to further investigate these pathways and inform culturally sensitive interventions aimed at reducing preterm birth disparities.

## Data Availability

Data are available from the corresponding author upon reasonable request.
